# MarA, RamA, and SoxS as Mediators of the Stress Response: Survival at a Cost

**DOI:** 10.3389/fmicb.2020.00828

**Published:** 2020-05-05

**Authors:** Emma R. Holden, Mark A. Webber

**Affiliations:** ^1^Quadram Institute Biosciences, Norwich, United Kingdom; ^2^Norwich Medical School, University of East Anglia, Norwich, United Kingdom

**Keywords:** efflux, biofilm, virulence, trade-off, regulation

## Abstract

To survive and adapt to changing environments, bacteria have evolved mechanisms to express appropriate genes at appropriate times. Exposure to antimicrobials triggers a global stress response in Enterobacteriaceae, underpinned by activation of a family of transcriptional regulators, including MarA, RamA, and SoxS. These control a program of altered gene expression allowing a rapid and measured response to improve fitness in the presence of toxic drugs. Increased expression of *marA*, *ramA*, and *soxS* up regulates efflux activity to allow detoxification of the cell. However, this also results in trade-offs in other phenotypes, such as impaired growth rates, biofilm formation and virulence. Here, we review the current knowledge regarding the trade-offs that exist between drug survival and other phenotypes that result from induction of *marA*, *ramA*, and *soxS*. Additionally, we present some new findings linking expression of these regulators and biofilm formation in Enterobacteriaceae, thereby demonstrating the interconnected nature of regulatory networks within the cell and explaining how trade-offs can exist between important phenotypes. This has important implications for our understanding of how bacterial virulence and biofilms can be influenced by exposure to antimicrobials.

## Introduction

**FIGURE 1:**
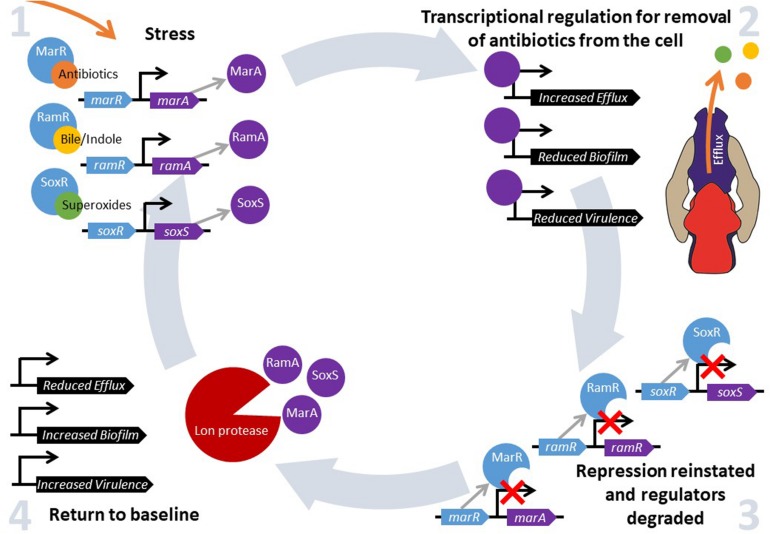
MarA, RamA and SoxS regulate fitness in response to environmental stress. Intracellular accumulation of a stressor is sensed by MarR, RamR, and/or SoxR, depending on the substrate. Subsequently, substrate binding to MarR, RamR, and SoxR prevents the repression of *marA*, *ramA*, and *soxS* expression, respectively. MarA, RamA, and SoxS activate transcription of a wide range of genes, which results in increased drug resistance. However, another set of genes are repressed in response, for example genes involved in biofilm formation and virulence. When intracellular concentrations of the inducer fall to basal levels, MarR, RamR, and SoxR are no longer inhibited and can bind to the promoter sequences of *marA*, *ramA*, and *soxS*, respectively, preventing their transcription. Pre-produced MarA, RamA, and SoxS are degraded by the Lon protease, and drug resistance, biofilm formation and virulence gene expression return to baseline levels.

Bacteria constantly have to adapt and evolve in response to changes in their environment. Changes in temperature, pH, oxygen and nutrient availability and exposure to antibiotics all require bacteria to respond by altering the expression of relevant protective genes in an effective and timely manner in order to survive (Dorman, 1996). This can be facilitated by the “general stress response”, where alternative sigma factors mediate a new program of gene expression, or by global stress responses, which control gene expression via “master” transcriptional regulators that are activated when a cell senses environmental change. These transcriptional regulators bind to and control gene expression to increase bacterial fitness in a challenging environment. There are many families of transcriptional regulators that respond to different environmental conditions. One subset control a drug protective response; MarA, RamA, and SoxS are members of the AraC/XylS family of transcriptional regulators found in Enterobacteriaceae (Gallegos et al., 1997). They are pleiotropic regulators that bind many sites across the genome and play an important role in antibiotic resistance through their influence on efflux activity. They also impact biofilm formation, quorum sensing, pathogenicity and motility (Duval and Lister, 2013). Each of these transcriptional activators is repressed under normal conditions by their cognate regulators *marR*, *ramR*, and *soxR*, which act by inhibiting expression of the activators *marA*, *ramA*, and *soxS*, respectively (Abouzeed et al., 2008; Duval and Lister, 2013). Changes in environmental conditions are sensed through substrates binding to these local repressors, thereby inactivating them and relieving repression of expression of *marA*, *ramA*, and *soxS* (Demple, 1996; Alekshun and Levy, 1999a; Abouzeed et al., 2008). When local repression is ablated, transcription of these regulators is autoregulated and stimulated by MarA, RamA, SoxS, which bind to the promoter regions and activate transcription (Alekshun and Levy, 1999b; Rosenblum et al., 2011). When the signaling substrate has been removed from the cell and *marR*, *ramR*, and *soxR* are no longer inhibited by environmental signals, repression is reinstated as MarR, RamR, and SoxR are able to bind to their regulatory DNA targets again. For example, it has been shown that there is a rapid increase in transcription of *marA*, *soxS* (Griffith et al., 2004) and *ramA* (Ricci et al., 2014) following exposure to antibiotics (and other inducer substrates), but that repression is rapidly reinstated following removal of the stimuli. The pool of pre-produced transcriptional regulators are degraded by proteases including Lon, and this “resetting” in impaired in *lon* deficient mutants (Griffith et al., 2004; Ricci et al., 2014). This ability to quickly produce, but then degrade, MarA, RamA, and SoxS allows for a fine-tuned, fast response to environmental stimuli to maximize bacterial fitness when under stress (Figure 1).

MarA, RamA, and SoxS activate overlapping regulatory networks in response to environmental changes, but can be induced by different stresses. The multiple antibiotic resistance (*mar*) operon is one of the main regulators of drug resistance in *Escherichia coli* (Alekshun and Levy, 1999b). MarA was first identified in *E. coli*, and homologs have since been found in many other species, such as *Burkholderia* spp., and *Yersinia pestis* [see Alekshun and Levy (1999b) for review] (Udani and Levy, 2006; Gupta et al., 2019). MarR represses the transcription of *marA* in the absence of induction (Cohen et al., 1993). MarR was first shown to be inhibited by salicylic acid (which then results in *marA* overexpression), but many drugs are now known to be ligands of MarR that bind to and initiate *marA* expression. Substrate binding causes a conformational change in MarR that prevents it from binding upstream of *marA* and subsequently repression of *marA* expression is released (Alekshun and Levy, 1999b; Perera and Grove, 2010). ChIP-seq analysis has characterized the *mar* operon and confirmed the role of MarA as a regulator of membrane permeability, as well as genes required for lipid trafficking and DNA repair (Sharma et al., 2017).

RamRA (resistance antibiotic multiple) is present in Enterobacteriaceae including *Salmonella*, *Klebsiella*, *Enterobacter*, *Citrobacter*, but not *E. coli* (George et al., 1995; Blair et al., 2014). In these bacteria, the *ram* operon appears to be the main regulator of antibiotic resistance, although the *mar* operon is also present. As well as antibiotics, RamR ligands include bile acids and indole, suggesting a role for survival in the gut. These substrates bind to RamR to prevent it from binding to the *ramA* promoter, thereby allowing *ramA* expression (Abouzeed et al., 2008; Nikaido et al., 2011; Baucheron et al., 2014; Yamasaki et al., 2019). RamA has also been implicated in the regulation of membrane permeability and regulation of some ribosomal, amino acid and LPS biosynthetic pathways (Bailey et al., 2010; De Majumdar et al., 2015). Whilst there are common features in the genes controlled by RamA between species, there are species specific differences, for example in the specific repertoire of virulence effectors influenced.

Transcription of *soxS* (superoxides) is upregulated when the cell is under oxidative stress (Demple, 1996). Its local regulator, SoxR, contains a [2Fe–2S] cluster that is inactivated when oxidized by superoxides, nitric oxides and paraquat, thereby allowing transcription of *soxS* (Fujikawa et al., 2012). As well as controlling membrane permeability through efflux pump and outer membrane porin expression, SoxS is important for initiating transcription of genes to reduce superoxide and nitric oxide stress in the cell (Demple, 1996).

Rob is another transcriptional regulator belonging to the AraC/XylS family, but is structurally and functionally distinct from MarA, RamA, and SoxS. Rob is constitutively expressed and always present in the cell in high quantities, whereas levels of MarA, RamA, and SoxS in the cell are low under basal conditions as described above (Bennik et al., 2000). Rob is regulated through a post-transcriptional “sequestration-dispersal” mechanism, where clustering of Rob prevents its C-terminal domain from binding to DNA and renders it inactive and non-functional, but also prevents its degradation by the Lon protease. When activated by an inducer, dispersal of Rob frees up the C-terminal domain to initiate transcription of target genes (Rosner et al., 2002; Griffith et al., 2009). Overexpression of *marA*, *soxS*, (Webber et al., 2005) and *ramA* (Rosenblum et al., 2011) has been identified in clinical isolates but not *rob* (Piddock, 2006a). It has been found that Rob has a moderate effect on transcription of target genes and needs to be overexpressed at very high levels in order to see a change in phenotype (Bennik et al., 2000). Due to these differences, Rob will not be discussed further in relation to adaptive stress responses.

As well as mediating a drug-tolerance phenotype, there is evidence that MarA, RamA, and SoxS also impact the expression of multiple genes involved in many different pathways. These include genes involved in bacterial pathogenicity and biofilm formation, highlighting the interconnected nature of diverse regulatory networks within the cell. This illustrates how trade-offs exist between important phenotypes.

## Control of Drug Accumulation: Efflux and Porins in Response to Stress

Efflux pumps are membrane-located transporter proteins that export toxic substances from the cell [See Du et al. (2018) for a review]. They are fundamentally important in exporting antibiotics from the cell and have been shown to confer decreased susceptibility to a wide range of commonly used antibiotics (Li et al., 1994; Blair et al., 2014). The most clinically important family of multidrug resistance efflux pumps in Enterobacteriaceae is the resistance nodulation division (RND) family, and more specifically, the AcrAB-TolC system (Blair et al., 2014). Homologs of AcrAB-TolC have been identified in many commensal and pathogenic bacteria [see Piddock (2006a) for review]. This pump is regulated by *marA*, *ramA*, and *soxS*, each of which can promote increased transcription of *acrAB* and *tolC* (Okusu et al., 1996). As well as AcrAB-TolC, MarA, RamA, and SoxS can also regulate the expression of other efflux pumps, such as the RND pump AcrEF (Bailey et al., 2010) and a member of the multidrug and toxic compound extrusion (MATE) family, *mdtK* (Sun et al., 2011) in response to environmental stress.

Upregulating efflux pump expression through overexpression of *marA*, *ramA*, and *soxS* is an efficient mechanism to allow detoxification of the cell of antimicrobial chemicals. Whilst efflux alone may only confer relatively modest changes in drug susceptibility, it has been shown to act as a platform for other resistance mechanisms. For example, target site mutations in *gyrA*, conferring fluoroquinolone resistance, do not result in clinical resistance when efflux is inactivated (Kern et al., 2000; Oethinger et al., 2000). This is also seen in *Campylobacter* spp., where ribosomal mutations conferred an increase in the MICs of erythromycin and tylosin, but inactivation of the AcrB homolog, CmeB, resulted in a drug-susceptible phenotype in isolates with these ribosomal mutations, with MICs below wildtype for both drugs (Cagliero et al., 2006). This demonstrates the clinical relevance of efflux activity.

Although changes in expression of multidrug efflux pumps will not often alone confer large changes in antibiotic susceptibility, changes in *marA*, *ramA*, and *soxS* expression are an important mechanism in isolates that demonstrate multidrug resistance (Abouzeed et al., 2008; Duval and Lister, 2013). In *S*. Typhimurium, mutations in the local repressors *marR*, *ramR*, and *soxR* are common in clinical isolates with reduced susceptibility to multiple drugs (Piddock, 2006a). Whilst there are clear overlaps in the phenotypes conferred by the genes controlled by MarA, RamA, and SoxS, there appear to be differences in the relative importance of each system in different species and in response to difference stresses. MarA has often been found to be the most important transcriptional regulator for conferring AcrAB-TolC-mediated drug resistance in *E. coli*. For example, fluoroquinolone stress selected for constitutive expression of *marA* through inactivation of *marR* in *E. coli* (Kern et al., 2000). Inactivation of *marA* in *E. coli* resulted in increased susceptibility to the organic solvent cyclohexane, but inactivation of *soxS* had no effect (White et al., 1997). However, inactivation of *marA* or *soxS* had no effect on drug susceptibility in *S*. Typhimurium when *ramA* was active, indicating that RamA is more important for drug efflux in *S*. Typhimurium (Abouzeed et al., 2008). RamA was also seen to be more important than MarA or SoxS in *Klebsiella pneumoniae*, where overexpression of *ramA*, but not the others, was found in response to tigecycline stress (Rosenblum et al., 2011). When *ramA* was inactivated in *K. pneumoniae*, increased expression of *marA* and another AraC-type regulator, *rarA*, was seen in response, which resulted in low-level multidrug resistance (Veleba and Schneiders, 2012). This demonstrates the redundancy in these regulators, as when one is inactivated, the others are often upregulated in response (Baugh et al., 2014). However, it is currently unclear how loss of function of one regulator is sensed and regulated within the cell. Despite differences between species, each transcriptional regulator retains its specificity to response stimulus, suggesting a shared evolutionary history. In *Enterobacter cloacae*, *ramA* was upregulated in response to sodium salicylate and tetracycline, but *soxS* was upregulated in response to paraquat (Pérez et al., 2012).

Control of efflux and sensitivity to cellular efflux function is a common theme for MarA, RamA, and SoxS. When efflux is disrupted, cells respond with overexpression of these transcriptional regulators, though it is not understood by which mechanism this is regulated. Overexpression of *marA*, *ramA*, and *soxS* were all seen when AcrAB-TolC efflux was inactivated (Webber et al., 2009; Zhang et al., 2017). Overexpression of *marA* and *ramA* was also seen following inactivation of other efflux pumps in *S*. Typhimurium, including *acrEF*, *acrD*, *mdsABC*, *mdtABC* (RND pumps), *macAB* (ATP-Binding Cassette (ABC) superfamily), *emrAB*, *mdfA* [Major Facilitator Superfamily (MFS)] and *mdtK* (MATE family) (Zhang et al., 2017). Chemical inhibition of efflux activity with phenylalanine-arginine beta-naphthylamide (PAβN) also resulted in increased expression of *marA* and *ramA* (Zhang et al., 2017). Western blotting has demonstrated that this increased transcription resulted in increased protein expression, where increased RamA was detected in *S*. Typhimurium when efflux was inhibited with chlorpromazine (Ricci et al., 2014). Additionally, overexpression of *marA, ramA*, and *soxS* was seen in *S*. Typhimurium following deletion of *csrA*, which encodes an RNA binding protein that controls transcript stability of *acrAB* mRNA (Ricci et al., 2017). Together, these studies show that any modulation of efflux pump expression or activity is sensed by the cell and causes overexpression of *marA, ramA*, and *soxS*. Our working model is that these regulators are overexpressed in response to intracellular accumulation of efflux substrates that trigger upregulation of efflux.

Membrane permeability is dependent on influx as well as efflux, therefore regulation of membrane porins goes hand in hand with efflux pump regulation for controlling susceptibility to antimicrobials. MarA, RamA, and SoxS regulate the transcription of *micF*, which encodes a small RNA that prevents the translation of a major membrane porin, OmpF (Cohen et al., 1988; Pomposiello and Demple, 2000; Zheng et al., 2011). Porin repression prevents influx of antibiotics, detergents and toxins into the cell, providing synergistic protection with enhanced efflux.

There is a trade-off between efflux pump expression and relative fitness, whereby increased pump expression is favorable under drug stress but detrimental in a neutral environment due to energetic costs (Wood and Cluzel, 2012). Mutations in the *marA* regulator *marR* and efflux pump regulator *acrR* that resulted in constitutive AcrAB-TolC pump expression could be selected for by ciprofloxacin exposure in *E. coli*, however, this conferred a fitness cost relative to the wild type when antibiotic stress was removed (Marcusson et al., 2009). Control of this system by global and local regulators allows the cell to balance the benefits and costs of expressing efflux pumps to maximize fitness at any given time in their life cycle. Recent research has reported that increased *marA* expression correlated with increased mutability in *E. coli*, and suggested that increased expression of *acrAB* led to decreased expression of the DNA mismatch repair gene *mutS*, decreased growth rate and an increased mutation frequency (El Meouche and Dunlop, 2018). However, an earlier study found no such link between mutation frequency and expression of *acrB* in *S.* Typhimurium under ciprofloxacin stress, where increased expression of *acrB* did not significantly change mutation frequency compared to wildtype *acrB* expression (Ricci et al., 2006). Work continues to try to understand the evolutionary trade-offs that exist between efflux activity and bacterial fitness, to maximize fitness in all environments. It is assumed that the metabolic burden of over-expressing efflux systems is responsible for a decrease in fitness, as found in *Stenotrophomonas maltophilia* over-expressing SmeDEF, homologous to the AcrAB efflux pump in *E. coli* (Alonso et al., 2004). However, this is not always the case, as no metabolic burden was observed in *Pseudomonas aeruginosa* when overexpressing the MexEF-OprN efflux pump, and the resulting reduction in virulence was suggested to be due to other changes in a global regulatory network (Olivares et al., 2012).

## Biofilms and Stress

Another important phenotype affected by *marA*, *ramA*, and *soxS* expression is biofilm formation. Most bacteria in nature are thought to exist in a biofilm; a structured community of bacteria aggregated together (Berlanga and Guerrero, 2016). Biofilms are clinically important, as approximately 80% of all infections have a biofilm component (Bjarnsholt et al., 2018). One of the hallmarks of bacteria found in a biofilm is their high degree of tolerance to a range of antibiotics, biocides, toxins and detergents. Changes in gene and protein expression result in low levels of metabolic activity and promote production of high numbers of persister cells. These are dormant, non-dividing cells that tolerate a wide range of antimicrobials, allowing biofilms to be typically 10-1000-fold less sensitive to drugs (Hoyle and Costerton, 1991; Mah et al., 2003). When grown in a biofilm, cells become intrinsically tolerant to antibiotics, and it is now thought that the main determinant of this is metabolic changes within the biofilm-forming cells, rather than structural features, that confer decreased drug susceptibility (Mah et al., 2003; Liu et al., 2018; Trampari et al., 2019). Chronic infections caused by biofilm-forming bacteria [for example, prosthetic joint infections or diabetic ulcers] are rarely resolved with antibiotic chemotherapy alone due to drug resistance, therefore biofilm infections can result in poor patient outcomes (Davis et al., 2006; Gbejuade et al., 2015). As well as being medically important, biofilms are also an important consideration for agriculture (Antunes et al., 2016), food processing environments (Kumar and Anand, 1998), water treatment processes (Schwartz et al., 2003), or any industry where bacterial decontamination is important.

As well as their role in drug resistance, global transcriptional regulators affect biofilm formation through a currently undefined relationship whereby inactivation or inhibition of efflux activity results in disruption of biofilm formation (Kvist et al., 2008; Baugh et al., 2012). This appears to be a result of an inverse regulatory relationship between efflux pump function and expression of biofilm matrix genes (Baugh et al., 2012, 2014). This seems evolutionarily counter-intuitive, as disrupting one mechanism of drug tolerance leads to the disruption of another. However, efflux upregulation in response to toxic stress may signal that the environment would be poor to permanently colonize, and therefore biofilm formation is repressed. The relationship between efflux activity and biofilm formation has been demonstrated in a wide range of bacteria, including *A. baumannii*, *E. coli*, *P. aeruginosa*, *S. aureus*, and *S*. Typhimurium [see Alav et al. (2018) for review].

The mechanism linking efflux and biofilm formation is unclear, however, no change in growth rate or cell surface hydrophobicity (which aids adhesion) could explain the link between biofilm formation and efflux activity (Baugh et al., 2012, 2014). There was also no evidence for quorum sensing molecules being exported via efflux to explain this relationship (Ahmer, 2004; Baugh et al., 2014). Disruption of efflux in *S*. Typhimurium was found to cause transcriptional repression of *csgB* and *csgD*, responsible for curli synthesis, which makes up a major component of bacterial biofilms. This suggests that the end biofilm deficit is a result of repression of curli biosynthesis in response to loss of efflux function (Baugh et al., 2012). One theory is that MarA regulates biofilm formation through binding upstream of the *ycgZ-ymgABC* operon, which has a role in curli formation (Kettles et al., 2019).

**FIGURE 2:**
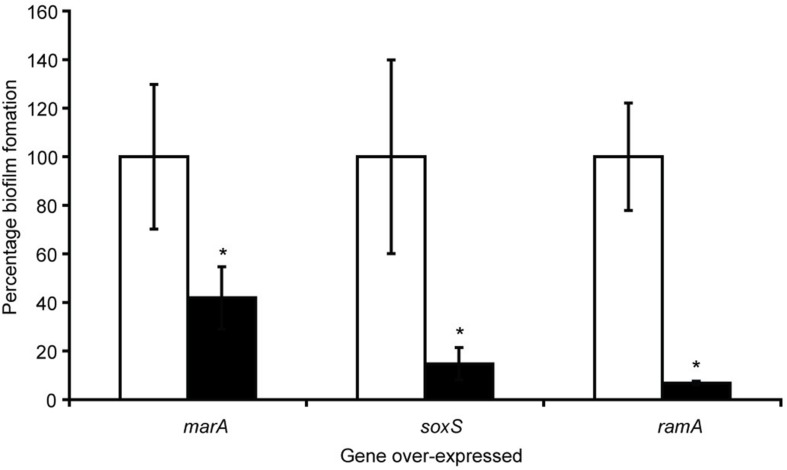
Biofilm formation as a percentage of the wild type, measured by a crystal violet biofilm assay [described by Baugh et al. (2012)], in *S*. Typhimurium 14028S transformed with pTrc-*marA*, pTrc-*ramA* and pTrc-*soxS*, when uninduced (white bars) and induced with 1 mM IPTG (black bars). Error bars represent 1 standard deviation and asterisks (*) represent statistically significant differences in biofilm formation between uninduced and induced treatments (*p* < 0.05) (Baugh, 2014).

**FIGURE 3:**
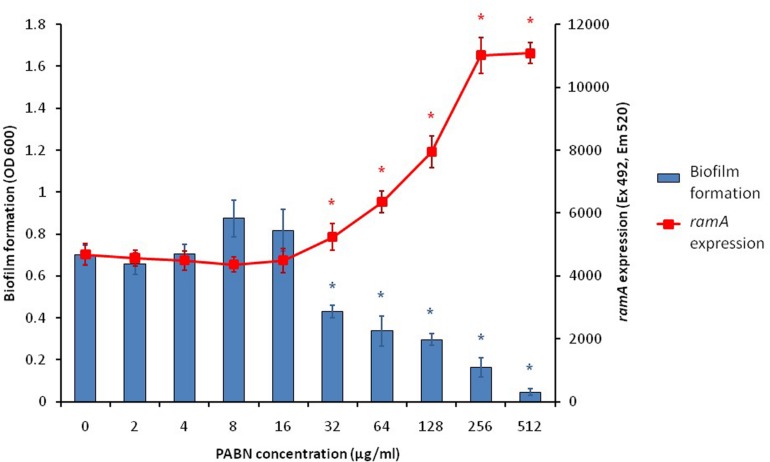
Biofilm formation (blue bars) and *ramA* expression (red line) in *S*. Typhimurium 14028S treated with increasing concentrations of the efflux inhibitor PAβN. Biofilm formation was measured using a crystal violet biofilm assay [described by Baugh et al. (2012)] and *ramA* expression was measured by cRT-PCR, following methods described by Eaves et al. (2004). Error bars represent one standard deviation and asterisks (*) show statistically significant differences in biofilm formation (blue) and gene expression (red) from the treatment without PAβN (*p* < 0.05) (Baugh, 2014).

With the knowledge that efflux inactivation results in overexpression of *marA*, *ramA* and *soxS* (Webber et al., 2009; Zhang et al., 2017), we sought to determine whether the deficit in biofilm formation was mediated by these transcriptional regulators. Using the expression plasmid pTrc, each transcriptional regulator was overexpressed in *S*. Typhimurium and their biofilm-forming ability was measured using a crystal violet biofilm assay. Artificially overexpressing either *marA, ramA* or *soxS* resulted in significantly reduced biofilm formation in *S.* Typhimurium (Figure 2; Baugh, 2014). This suggests that the increase in *marA, ramA*, or *soxS* expression following efflux inactivation may be behind the decrease in biofilm formation. We followed up this experiment by measuring how incremental addition of the efflux inhibitor PAβN, simulating gradual efflux inactivation, affected expression of *ramA* and biofilm formation (as expression of *ramA* is greatly increased in efflux deficient mutants of *S*. Typhimurium). We saw a dose-dependent increase in *ramA* and concomitant decrease in biofilm biomass (Figure 3; Baugh, 2014). This supports the idea that biofilm formation is sensitive to efflux function through the expression of *marA, ramA*, and *soxS*.

**FIGURE 4:**
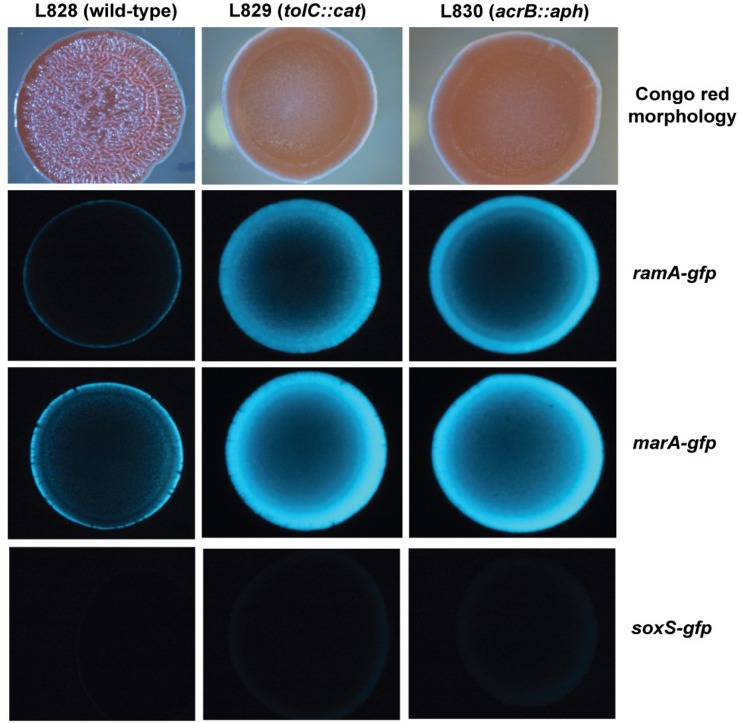
Congo red morphologies of wild type *S*. Typhimurium strain 14028S and two pump knockout strains *(tolC::cat* and *acrB::aph*) transformed with the *gfp* reporter plasmid pMW82 containing the promoter region of *marA*, *ramA* or *soxS* to demonstrate spatial expression of these regulators (Baugh, 2014).

To investigate spatial expression of these transcriptional regulators in the biofilm, we cloned the promoter regions of *ramA* and *marA* into the *gfp* reporter plasmid pMW82 and transformed into wild type *S*. Typhimurium and efflux-deficient mutants *tolC::cat* and *acrB::aph* (Baugh, 2014). Figure 4 shows the inverse relationship between curli expression and the expression of *ramA* and *marA*, where curli is expressed in the center of the colony and the transcriptional regulators are expressed at the perimeter. Colonies were plated on agar supplemented with Congo red, which is a dye that binds to curli in the biofilm to form a red, dry and rough morphology (rdar), as seen for the wild type. Pump knockout mutants have lower curli expression, shown on Congo red plates through the smooth and pale colony morphology. These colonies have a visible ring separating the center from the perimeter, showing the stationary-phase curli-producing cells at the centre of the colony and the growing cells at the perimeter of the colony producing less curli. Expression of *marA* and *ramA* occurs most in growing cells, seen in Figure 4 by the high GFP signal in growing cells round the perimeter of the colonies. Taken together, the data presented in Figures 2–4 demonstrate a clear relationship between biofilm formation and the expression of *marA*, *ramA*, and *soxS*, with an inverse relationship between regulator expression and curli biosynthesis. However, the pathway through which this is regulated is still unclear.

There is some evidence that c-di-GMP may play a role in the relationship between efflux activity and biofilm formation. This is a secondary messenger molecule that has been closely linked to the biosynthesis of two important components of the bacterial biofilm, cellulose and curli, although the mechanism by which c-di-GMP affects curli biosynthesis is unknown (Barnhart and Chapman, 2006). The deletion of *soxS* in *Klebsiella pneumoniae* caused a decrease in *yjcC*, which is a phosphodiesterase specific for breaking down c-di-GMP, suggesting that *soxS* activates *yjcC* (Huang et al., 2013). Our group conducted a transposon mutagenesis experiment in *S*. Typhimurium, which found that interrupting the activity of *yjcC* rescued biofilm formation when the EmrAB efflux pump had been knocked out, further supporting a possible link between efflux activity and biofilm formation (Baugh, 2014). A similar relationship is also seen in *K. pneumoniae*, where deletion of *yjcC* resulted in a significant increase in biofilm formation and increased production of MrkA, which is important for *Klebsiella* biofilms (Huang et al., 2013). However, when efflux activity is disrupted, cellulose biosynthesis remains unchanged (Baugh et al., 2012). This means that if c-di-GMP is important in the relationship between efflux activity and biofilm formation, it must be mediated through a pathway separate from that of cellulose biosynthesis. As well as *marA*, *ramA*, and *soxS*, modulation of these secondary messenger molecules also affects many aspects of bacterial behavior, such as the organism’s performance during infection.

## Pathogenicity and Stress Response

Bacterial pathogenicity or virulence can be defined in many ways, but here it will refer to disease severity. In evolutionary theory, there is a school of thought that dictates that pathogens should evolve toward commensalism (Alizon et al., 2009). It can be detrimental to the long-term survival and proliferation of a pathogen to damage and kill its host organism before its dissemination, therefore a lower virulence potential should allow for neutral coexistence between host and microbe. However, evolution is a random process and the trade-off between maximizing resource use and prolonging the life of the host environment is under ongoing selective pressure (Jansen et al., 2015). Due to their multiplicity of targets, it is understandable that manipulation of *marA*, *ramA* and *soxS* might affect pathogenicity, however, the pathways through which they act are not clear. When *soxS* or *rob* were inactivated in *E. coli*, mutants could not sustain colonization in a murine kidney model (Casaz et al., 2006). Attenuated virulence of *S.* Typhimurium in a *C. elegans* infection model was seen when *ramA* was either overexpressed or inactivated: survival in and adhesion to mouse macrophages was seen following inactivation of *ramA*, but worsened when *ramA* was overexpressed (Bailey et al., 2010).

Multidrug efflux pumps have been found to be important in bacterial pathogenicity, and in some cases are essential for causing infection (Piddock, 2006b). Similar to biofilm formation, there seems to be a trade-off between expression of efflux pumps and bacterial pathogenicity determinants, as demonstrated by attenuation of *S*. Typhimurium in a mouse model when various efflux systems were inactivated (Nishino et al., 2006). Whilst there is a clear impact on virulence from loss of efflux, the mechanisms underpinning this are not well understood. However, there is often a marked change in the regulators of efflux when efflux function is compromised. It has been shown that *marA*, *ramA*, and *soxS* are upregulated when efflux is disrupted, and it has been suggested that changes in virulence in response to loss of efflux may be mediated by changes in expression of these global transcriptional regulators that influence virulence genes. For example, when efflux was disrupted through deletion of *acrB* in *S*. Typhimurium, expression of *ramA*, *marA*, and *soxS* significantly increased and virulence was attenuated (Blair et al., 2015). Despite efflux being essential for virulence in some systems, this is not always the case. Following overexpression of *ramA* in *S*. Typhimurium, increased expression of *acrAB*, *acrEF* and *tolC* was observed, but this resulted in decreased adhesion to and survival in macrophages, as well as reduced virulence in a *C. elegans* model (Bailey et al., 2010). A molecular mechanism explaining this reduced pathogenicity has been elucidated in *K. pneumoniae*, where overexpression of *ramA* was found to activate lipid A biosynthesis and therefore modulate LPS biosynthesis, which plays a key role in host-pathogen interactions (De Majumdar et al., 2015). Given the fact that many of the members of the regulons of these transcriptional regulators are surface expressed proteins it is likely this may explain how their modulation would affect adhesion and therefore virulence through bacterial surface modification.

In *Salmonella enterica*, *marA*, *ramA*, and *soxS* have been shown to affect pathogenicity through controlling the expression of dedicated virulence factors, the *Salmonella* pathogenicity islands (SPIs). These encode secretion systems mediating pathogenicity and were acquired by horizontal gene transfer. Acquisition of these pathogenicity islands is thought to reflect the divergence of *S. enterica* and *E. coli* approximately 140 million years ago (Wirth et al., 2006). Two SPIs are essential for pathogenesis: SPI-1 contains genes that encode a type III secretion system essential for intracellular invasion, and expression of genes on SPI-2 allows for bacterial survival once inside macrophages (Boddicker and Jones, 2004). Overexpression of *ramA* was found to result in decreased expression of SPI-1 and SPI-2 virulence effectors and regulators (Bailey et al., 2010). A study in a *C. elegans* infection model found that cross-talk between SPI-1 and SPI-2 was essential for biofilm formation, which resulted in prolonged asymptomatic carriage and reduced virulence. A mutant lacking *ssrB*, a SPI-2 encoded transcriptional regulator, could not form biofilms *in vivo* and caused reduced host survival (Desai et al., 2019). This suggests that virulence and biofilm formation are controlled through similar regulatory networks, with both being influenced by MarA, RamA, and SoxS.

Motility can be important in bacterial virulence, insofar as it dictates how well a pathogen can spread to cause infection (Josenhans and Suerbaum, 2002). Downregulation of motility is also important in the switch from a planktonic to a biofilm-associated lifestyle (Rossi et al., 2018). Inactivation of efflux activity has been seen to reduce motility in *Acinetobacter nosocomialis* following the deletion of the *acrB* homolog, *adeJ* (Knight et al., 2018). Inactivation of *acrB* or *tolC* in *S.* Typhimurium resulted in significantly lower expression of genes involved in anaerobic metabolism, motility and chemotaxis (Webber et al., 2009). However, a different study showed that motility can be significantly improved through the deletion of *acrEF*, *mdsABC*, *mdtABC*, or *mdfA* efflux pumps in *S*. Typhimurium, and that this increase could be prevented through inactivation of *soxS* (but not *marA* or *ramA*) (Zhang et al., 2017). This suggests that motility may be regulated by the effect of *soxS* on c-di-GMP levels, as the secondary messenger molecule is known to bind to YcgR and negatively regulate flagella motor rotation (Paul et al., 2010). C-di-GMP also regulates biofilm formation through inducing cellulose biosynthesis, which is known to affect motility. When cellulase activity was inactivated in *S.* Typhimurium, flagella-based swimming and swarming motility was downregulated (Ahmad et al., 2016). This may be because increased cellulose production is indicative of a move toward a more static lifestyle in a biofilm where motility is not advantageous, and this is associated with reduced pathogenicity.

## Conclusion

Global stress responses control multiple phenotypes within the cell. Changes in gene expression attempt to maximize bacterial fitness in response to challenging environmental conditions, but trade-offs can have consequences on disease pathology. The family of regulators that include MarA, RamA, and SoxS are key in controlling the cell’s response to antibiotic stress, but also influence various other important phenotypes. With the increasing global incidence of antibiotic resistant pathogens, understanding how bacteria adapt and evolve to drug stress is extremely important. Experimental evolution has demonstrated through exploiting these trade-offs that decreased drug susceptibility can lead to lower pathogenicity, however, further studies are needed to determine whether this holds true *in vivo*. This has implications for treatment of bacteria in clinical settings, food processing environments, agricultural industries, water treatment facilities and many more environments where selection for antimicrobial resistance is known to occur. Understanding these complex regulatory networks in different conditions will be key to exploit regulatory trade-offs and the development of strategies to rationally alter bacterial behavior in beneficial ways.

## Data Availability Statement

The raw data supporting the conclusions of this article will be made available by the authors, without undue reservation, to any qualified researcher.

## Author Contributions

EH and MW wrote and edited the manuscript.

## Conflict of Interest

The authors declare that the research was conducted in the absence of any commercial or financial relationships that could be construed as a potential conflict of interest.
